# Picking and Choosing Among Phase I Trials: A Qualitative Examination of How Healthy Volunteers Understand Study Risks

**DOI:** 10.1007/s11673-019-09946-w

**Published:** 2019-11-12

**Authors:** Jill A. Fisher, Torin Monahan, Rebecca L. Walker

**Affiliations:** 1 Department of Social Medicine and Center for Bioethics, University of North Carolina at Chapel Hill, CB 7240, Chapel Hill, NC 27599-7240 USA; 2 Department of Communication, University of North Carolina at Chapel Hill, CB 3285, Chapel Hill, NC 27599-3285 USA

**Keywords:** Healthy volunteers, Phase I clinical trials, Risk perceptions, Informed consent, Decision-making

## Abstract

This article empirically examines how healthy volunteers evaluate and make sense of the risks of phase I clinical drug trials. This is an ethically important topic because healthy volunteers are exposed to risk but can gain no medical benefit from their trial participation. Based on in-depth qualitative interviews with 178 healthy volunteers enrolled in various clinical trials, we found that participants focus on myriad characteristics of clinical trials when assessing risk and making enrolment decisions. These factors include the short-term and long-term effects; required medical procedures; the type of trial, including its design, therapeutic area of investigation, and dosage of the drug; the amount of compensation; and trust in the research clinic. In making determinations about the study risks, participants rely on information provided during the consent process, their own and others’ experiences in clinical trials, and comparisons among studies. Our findings indicate that the informed consent process succeeds in communicating well about certain types of risk information while simultaneously creating lacunae that are problematically filled by participants through their collective experiences and assumptions about risk. We discuss the ethical implications of these findings and make recommendations for improving the consent process in healthy volunteer trials.

## Introduction

In clinical trials, the informed consent process has many objectives, but crucial among them is sharing information about risks of study participation in a manner that is meaningful to potential participants. As an ethical standard, the information provided should give a sufficient basis for individual decision-making regarding the reasonableness of trial risks (Faden and Beauchamp 1986). At the same time, we know that prospective participants bring to bear their own medical histories, knowledge about health and illness, and motivations to enrol in clinical trials (e.g., Kent 1996; Siminoff, Caputo, and Burant 2004; Fisher 2009). Risk perceptions are also shaped by individuals’ broader views of medical research, the institution conducting the clinical trial, and the possibility for medical benefit (e.g., Lidz et al. 2004; Corbie-Smith, Thomas, and St. George 2002; Kingori 2015). Given this reality, it is important to explore empirically how risk perceptions precede and mediate formal informed consent processes and how they guide individual decision-making.

Healthy volunteers who participate in phase I clinical trials offer a case study of how risk perceptions are constructed, negotiated, and maintained. Healthy volunteers are a particularly important population of clinical trial participants because they have no possibility for direct medical benefit that might offset the risk—or their perception of the risk—to which they are exposed. Specifically, phase I trials test the safety and/or tolerability of investigational drugs. These trials help establish doses for future trials and assess a medication’s adverse effects rather than test its efficacy (Kass et al. 2007). Bioethics scholars generally consider these trials to be low risk (e.g., Emanuel et al. 2015; Johnson et al. 2016), but participants routinely experience temporary discomforts, such as headaches and gastrointestinal changes, as well as more serious issues, such as changes to kidney or liver function and to blood counts or production, which generally resolve quickly after discontinuation of the investigational drug (Sibille et al. 1998). Prospective participants are nonetheless warned that serious or life-threatening problems can occur, including the possibility of death (Chan 2016; Wood and Darbyshire 2006). Published studies show that healthy volunteers appraise risk when deciding whether to enrol in phase I trials (Roberts and Kim 2017; Grady et al. 2017). Yet, little is known about the particular factors that inform healthy volunteers’ risk perceptions.

Phase I trials are also distinct from later-phase trials in two important ways that might influence healthy volunteers’ risk perceptions. First, participants are often confined to a residential research facility during phase I trials. This requirement standardizes participants’ diet and activity, and it allows for frequent data collection through blood draws and other medical procedures (Fisher 2020). Second, most healthy volunteers enrol serially in phase I trials for the financial compensation, a practice which makes them quite savvy research participants (Abadie 2010; Elliott 2008; Tishler and Bartholomae 2003). Confinement and repeat participation shape healthy volunteers’ risk perceptions through their personal trial experiences (Fisher 2015a) and the stories they share with each other about risky studies (Fisher 2015b). These factors together imply that informed consent processes—or ethical positions—that presume a “study naïve” population (Fisher 2006; Grady 2015) may be delusory in phase I trials. To achieve sufficient contextual grounding for adequate consent in these particular circumstances, one must attend to healthy volunteers’ actual formation of phase I trial risk perceptions. Doing so can help researchers assess how well they are succeeding in fulfilling the ethical ideal of meeting participants where they are in the informed consent process.

Drawing upon in-depth qualitative research with 178 U.S. healthy volunteers, this investigation aims to illustrate potential ethical complexities in how participants with a range of trial experiences understand risk when they enrol in clinical trials for compensation. We find that myriad factors shape how healthy volunteers make determinations about the risks as part of their decisions to enrol in phase I trials. Our findings illustrate how the informed consent process successfully communicates certain types of risk information while simultaneously creating lacunae that participants problematically fill by drawing on their collective experiences and assumptions about risk. These gaps in the informed consent process, moreover, are not obviously perceptible to the ethically concerned researcher or ethics review board member. Such blind spots in the ethical conduct of phase I trials require attention to the perceptions of healthy volunteers themselves.

## Methods

This article uses a single wave of data from a longitudinal study on healthy volunteers’ phase I participation (Edelblute and Fisher 2015). To identify clinical trial participants, we recruited our sample from seven phase I clinics across the United States (three on the East Coast, two in the Midwest, and two on the West Coast). This clinic sampling strategy, which we deployed successfully in previous research (e.g., Fisher 2015a), was designed to increase the demographic diversity of healthy volunteers in our study. The clinics were not involved in the study design or data analysis; they merely gave us permission to recruit their trial participants for our study. The phase I trials in which participants were enrolled varied from clinic to clinic, with our approach targeting healthy volunteers broadly rather than specific types of phase I trials or therapeutic areas. All healthy volunteers who spoke either English or Spanish and were enrolled in a clinical trial during our clinic recruitment visits were eligible to participate in our study. Approximately 90 per cent of the participants we invited to participate enrolled. The study was reviewed and approved by the Biomedical Institutional Review Board at the University of North Carolina at Chapel Hill. All research participants provided written informed consent.

For this article, we draw upon the semi-structured interviews we conducted with 178 participants between May and December 2013 at the time of their enrolment in our larger study. Semi-structured interviews follow an interview guide but allow the interviewer to adapt the questions asked of participants based on each individual’s responses and experiences. This interviewing method is particularly well suited to exploratory research in which interviewers want to leave the range of responses to questions as open-ended as possible (Patton 2002). These interviews were conducted face-to-face, and they probed participants’ perceptions of the risks and benefits of trial participation, their decision-making about enrolment in trials, and their prior experiences in studies. We also collected participants’ demographic information, such as their gender, race, ethnicity, employment status, and household income.

Table 1 provides our sample’s demographic characteristics. Reflecting broader trends in healthy volunteer trials (Grady et al. 2017; Fisher and Kalbaugh 2011), our participants were predominantly men (74%) and from underrepresented minority groups (68%). The typical participant was un- or underemployed, had an annual household income of less than $25,000, did not have a college degree, and was over the age of thirty. The majority of participants also had prior phase I trial experience. Only 21 per cent were in their first clinical trial when we enrolled them in our study, and more than half (51.1%) had participated in at least five studies.

**Table 1:** Demographics of study participants (n = 178)

	n	%
**Gender**		
Women	47	26.4%
Men	131	73.6%
**Race/Ethnicity**		
Non-Hispanic white	57	32.0%
Black / African American	72	40.4%
American Indian	2	1.1%
Asian	6	3.4%
Hawaiian / Pacific Islander	2	1.1%
More than one race	13	7.3%
Hispanic^1^	38	21.3%
**Age**		
18–21	6	3.4%
22–29	34	19.1%
30–39	58	32.6%
40–49	54	30.3%
50+	26	14.6%
**Household Income**^**2**^		
Less than $10,000	30	16.9%
$10,000 to $24,999	52	29.2%
$25,000 to $49,999	71	39.9%
$50,000 to $74,999	13	7.3%
$75,000 to $99,999	7	3.9%
$100,000 or more	4	2.2%
**Educational Attainment**		
Less than high school	12	6.7%
High school or equivalent	37	20.8%
Some college	52	29.2%
Trade/technical/vocational training	19	10.7%
Associate degree	21	11.8%
Bachelor’s degree	32	18.0%
Graduate degree	5	2.8%
**Employment Status**^**3**^		
Full-time / business owner (self-employed)	45	25.3%
Part-time / independent or irregular contractor	60	33.7%
Unemployed / retired	73	41.0%
**Clinical Trial Experience**		
1 study	38	21.3%
2–4 studies	49	27.5%
5–10 studies	45	25.3%
11–200 studies	46	25.8%

^1^The category Hispanic includes all racial groups. Hispanic participants in our sample identified as white, black, American Indian, Native Hawaiian/Pacific Islander, and more than one race.

^2^Datum for household income was not reported by one participant.

^3^These data are based on consolidated definitions of each employment category that we used to standardize self-reported data from participants.

All interviews were transcribed in full, and two members of our research team coded each transcript using Dedoose qualitative research software. The codebook captured themes under investigation in the study (e.g., risk perception, benefit perception, decision-making) as well as themes that emerged from participants’ interviews (e.g., specific study risks, study oversight as a risk mitigator, trust in the clinics). As part of our analysis, we exported from Dedoose all transcript excerpts that had been coded as risk perceptions. Reading through the 2,018 risk-related excerpts from the 178 participants, we then created a list of factors that contributed to participants’ determinations of risk, identifying when participants were relying on the consent process, information gathered through their clinical trial experience or other participants, and/or a misunderstanding of the risks. We then identified representative quotes for the most salient of these factors. When presenting quotes below, we use pseudonyms to maintain the confidentiality of our participants’ identities.

## Results

Healthy individuals interested in enrolling in a phase I trial often have the opportunity to pick and choose among a variety of different studies. Even a single research facility might open recruitment for several trials simultaneously. As a result, prospective participants could decide to join a study based on its logistics, such as the dates or length of confinement, the amount of compensation, and/or the risks. Here, we concentrate on how healthy volunteers make sense of phase I trial risks, particularly when making decisions about their own study participation. We do so by investigating how risk perceptions are shaped both by information traditionally included in and absent from the trial consent process, as well as personal factors, such as family medical histories and trust in the research enterprise.

### Comparative Risk Evaluations

Even though compensation motivates healthy volunteers to enrol in phase I trials, risk assessment also affects their decision-making. However, participants offer different comparative frameworks for their assessment of trial risks. For example, Willie, a black man in his thirties who had participated in six studies, weighed the financial benefit against the risk:

It’s good paying rewards, but … there’s always a consequence or something that’s bad could happen. So, I’d have to be mindful of those risks. … Money is not really everything in life, you know. … Health is your greatest asset.

Participants adamant about the value they place on their health must judge which studies are worth the offered compensation. Leon, a black man in his thirties who had participated in fifteen studies, explained,

Some studies you would feel like, okay, … it’s not worth just the risks, you know. … [To make a decision] I might as well know exactly what’s going on, what the medication is, how many times it’s been tested on humans—not lab rats, not monkeys, not dogs. … I wanna know how many milligrams it is, how many times I will be dosed, you know, so that’s something that you have to look out for if you ever plan on doing studies.

Leon relied on a lay form of scientific expertise about phase I trial risks to determine which studies to join or avoid. As another example, Victor, a black man in his forties who had participated in an estimated seventy studies, believed that phase I participation is inherently risky, so he based his decisions on the severity and duration of possible side effects:

There’s going to be risk. … The question now is what … kind of studies do I want to do? The specific studies that I wouldn’t do: anything that has to do with my nervous system, I wouldn’t touch. If it’s a-, let’s say, hep C, there is a chance it’s going to pass through my body real quick. You know, I’ll find out what the half-life is and just how much we’re gonna get dosed. So, you weigh how much you getting dosed and weigh how much you getting paid, and then you compare that to the side effects. … Because if the side effects was extremely bad, I wouldn’t even do it.

While the dose of the drug was also important to Victor, he focused more on the class of the investigational drug, the time expected for it to remain in his body, and the potential side effects. Importantly, all three men claimed that they would refuse certain studies regardless of the compensation. As we explore below, healthy volunteers weigh myriad factors as they choose among studies with differing types of risk.

### Short-Term Effects

The key risk information included in consent forms and trial information sheets pertains to the potential side effects of the investigational drug. In phase I trials, these are typically short-term symptoms that are expected to resolve after discontinuation of the drug (Johnson et al. 2016). When the possible side effects mirror everyday ailments, healthy volunteers tend to voice less concern about the risks of participating. Symptoms perceived as unusual or extreme are more likely to evoke concern, whether or not these are literally detailed during the consent process. For example, Rubin, a white man in his twenties enrolled in his first study, commented on that trial’s risks:

Like the side effects they have for this one—nausea, headache, fever, flu-like symptoms—that’s not very much. [In the past,] I had the flu for a week, and I was stuck on the couch and I was puking and using the bathroom every ten minutes. That was-, [pauses] that I could deal with [in a study]. But [if it were] anything like malfunction with my body or like start losing parts, [*laughs*] stuff like that, no, I wouldn’t do it.

Another first-time participant, Jennifer, a white woman in her twenties, was similarly nonchalant about the risks, saying, “[The] side effects it had weren’t really that horrible, so that didn’t worry me.” While she did not welcome symptoms like headaches, she saw these as “normal” risks of any prescribed medication. When thinking about the risks that might have dissuaded her from enrolling in a clinical trial, she identified some that would be unacceptable to her:

Anything that like actually changes your organs, you know, like would enlarge your spleen. I don’t know if I would be willing to do something where it affected blood clotting ‘cause I would be afraid that I would have an aneurism or like a bruise that just caused me to bleed out. You know, when there’s like any real risk that you could die from something, I don’t think that’s worth it [to participate], you know.

Both Rubin and Jennifer downplayed side effects that mirrored conditions they might have outside of a clinical trial, but they noted that hypothetical life-threatening risks would limit their trial participation.

Risk information about potential side effects is not limited to what healthy volunteers read in a consent form or trial information sheet. In Jennifer’s case, the risks she deemed too extreme to accept, while sounding somewhat random, were based on conversations she had with other participants during her clinical trial. The clinic at which she was enrolled was simultaneously testing a blood thinner in another group of healthy volunteers, and several participants in her study had completed a prior clinical trial for a leukaemia drug in which they reported having experienced painful effects on their spleen. For first-time participants in particular, the experiential information they receive from other healthy volunteers helps them to contextualize the broader risks of phase I trials. Healthy volunteers might also request more information about side effects from the clinic staff during the informed consent process. Charlie, a white man in his forties who had participated in sixty studies, discussed the difference between official study documents and what one can glean from the staff. For the trial in which he was enrolled at the time of the interview, he remarked,

They had quite [a] fairly extensive list [of side effects], but I knew we weren’t going to see any of them. … When I was consenting, she [the staff member] said, “Oh, we studied this drug, and … those side effects there are listed for-, you know, they happened when they were at the higher doses [of the drug].” She goes, “You guys aren’t going to see anything.” It was straight out of the, you know, the horse’s mouth, actually.

Not only can such contextually informed disclosures diminish participants’ sense of risk, but they can also prepare participants for unpleasant symptoms, thereby making them feel fully aware of what they are getting themselves into. Harrison, a white man in his forties who had participated in four studies, illustrates this case. In one of the studies he had joined, the staff had warned participants to expect localized pain during an intravenous infusion of the investigational drug. He recalled,

It caused quite a bit of pain. … And they sort of knew that going in. I was the—I want to say—third cohort, and they upped it [the dose of the drug] a little bit each time. … So, I knew it was coming, they told me it was coming, so I could brace for it.

Knowing information in advance about particularly painful or difficult events likely to occur in the course of a study might motivate some people to decline participation. For those who do enrol, their experience of those side effects is often tempered by knowing the symptoms will be short-lived.

### Types of Trial Design

Phase I trials are designed to measure different aspects of the safety of investigational drugs, and they include first-in-human trials, ascending dose trials, and drug–drug interaction trials, to name a few. Without receiving any explicit comparison of these trial designs, healthy volunteers form their own judgments about how risks might differ. As one common example, many healthy volunteers have enrolled in bioequivalence studies. Unlike phase I trials of an investigational drug with limited safety data, bioequivalence studies compare an FDA-approved drug to a generic copy of that drug to prove that the generic is absorbed, metabolized, and excreted similarly to the original drug. Healthy volunteers are still exposed to side effects from these drugs, but many participants perceived the risks to be much lower than studies testing an investigational drug. For instance, Eugene, a white man in his forties participating in his second study, said, “I had no major concerns [about this study]. I was really, you know, quite at ease because it’s already a marketed drug, so I felt there was a lot less risk involved than me testing an investigational drug.” Similarly, Lauren, a white woman in her forties and in her second clinical trial, was also participating in a bioequivalence study. Thinking about the risk, she declared, “The medicine we’re being injected with is an FDA-approved drug, so I didn’t feel scared. I mean, I don’t feel like I’m a guinea pig or anything.”

Even with trials of investigational drugs, participants distinguished between the risks of different trial designs. In first-in-human (FIH) dose-escalation studies, some believed it is safer to participate early in the trial at the lowest dose, whereas others asserted that being in the first groups of humans to be dosed is riskier. Mindy and Jesse represent these two views of FIH trials respectively. Mindy, a white woman in her fifties in her second trial, opined,

I think we’re the first cohort for this drug, which is always if I got in on a study, I would want to get in [the] first cohort where the drug is just at the beginning because, you know, I would fear damage of [*sic*: from] higher doses of drugs. … That’s the only way I would do it.

Jesse, a Hispanic man in his thirties, had participated in ten studies and took a different stance on FIH trials:

Like, if it’s the first in humans, those are the ones where I’m kinda like concerned about, but if I see, like, I’m the second or third cohort, then I’m a little bit more at ease ‘cause there’s another group that took it before me and they list the side effects on the consent form, what they [the other groups] experienced.

The case of FIH studies illustrates how the same risk information conveyed to healthy volunteers can be understood and processed in divergent ways, even leading individuals to make different decisions about which trials to join or avoid.

### Study Procedures

Healthy volunteers also differentiate among phase I risks based not on the drug being tested but on the medical procedures required. While blood and urine collection and ECGs are fairly standard in phase I trials, some studies include more invasive tests, such as muscle biopsy, lumbar puncture, or bronchoscopy (Dominguez et al. 2012). These procedures alone have prompted healthy volunteers to decline studies. Mauricio, a Hispanic man in his twenties who had participated in four studies, recalled a study he had been offered that included a muscle biopsy. Not even remembering the medication being tested, Mauricio described his refusal to participate:

When they told me what it was, that they are going to cut out a piece of my leg like that to test something, … I said, “Really, no,” I said no. [*laughs*] “We’re grateful [for the opportunity], [but] I’m going to wait for another [study].” … I really did not want that. … Because the truth is I don’t know why really [they would do a biopsy]. They [usually] take your blood, but to me that seems like normal or something. I don’t know, but to rip out a piece of the body? No, really no. (*translated from Spanish*)

With this clinical trial, Mauricio worried more about the violence a biopsy would do to his body than about the drug itself. Although a biopsy would certainly be uncomfortable, it is uncertain that its risk would be greater than the drug being tested.

A similar phenomenon occurs with lumbar puncture studies. Lumbar punctures have serious risks, such as the commonly occurring spinal headache, which results from decreased pressure in the brain after cerebrospinal fluid collection, or the rarer possibility of infection, bleeding, longer-term back or leg pain, or brain herniation (Cavens and Ramael 2009). Participants primarily feared they could be paralyzed if the physician made a mistake during the procedure. This anxiety comes across in how Colton, a black man in his forties who had participated in twenty studies, portrayed the procedure:

I seen a study at a place [in the Northeast]. And I was going to do it, but it said it was a lumbar puncture, and I was like, “I’m not doing that ‘cause that’s my spine. [If] Somebody has a bad day [or] I get scared and jump, I’m paralyzed from the waist down from a study for $3,000. Nah, it’s not worth it.”

Phase I trials might include a lumbar puncture because the pharmaceutical company developing the drug is investigating whether it crosses the blood–brain barrier, which might be the desired mechanism of action for therapeutic areas like Alzheimer’s disease (Pardridge 2009). Nonetheless, participants generally were not focused on drug risks—and what it might mean for an investigational substance to be present in their brain and central nervous system. This was equally true for those participants who had consented to and participated in lumbar puncture studies and, therefore, might have had a better grasp on these trials’ actual risks. Liam, a white man in his twenties, had participated in thirteen studies, nine of which had required at least one lumbar puncture. Although he rated phase I trial risks as a four or lower on a ten-point scale, he placed studies with lumbar punctures at a seven or eight, reasoning:

I’ve never had any complications, but, I mean, you are puncturing like a major part of your body, and that’s your spinal column system. … You’re depressurizing it, and it’s invasive, so you’re opening your body up to getting infections and things like that. So, but I feel like they know how to do the job, and they do it well, to the point where the risk for those things happening is very low, even though the risk still is there. So, I wouldn’t like completely rate it at the very top [of the risk scale], but I’d say, you know, with it being so invasive, yes [it’s a seven or eight].

In discussing the lumbar puncture studies he had done, Liam dismissively mentioned they were phase I trials for antidepressants, implying the study drugs raised no concern about risk at all. In short, some procedures may dominate participants’ risk perceptions such that the drug risks are diminished or not brought to bear on their trial decision-making.

Overall, participants have had mixed results undergoing biopsies and lumbar punctures, and negative information about these studies travels fast among healthy volunteers. Given that these studies are conducted in the same clinics at the same time as other phase I trials, healthy volunteers could witness how others undergoing these procedures fare. In addition, invasive procedures make for particularly compelling tales when participants swap “war stories” about their prior clinical trial exploits (Fisher 2015b). Vicarious experiences of these procedures, even when exaggerated, become an important source of risk information for many participants. For Lena, a Hispanic woman in her fifties who had participated in fifteen studies, a muscle biopsy had not sounded very risky, but her perceptions changed after seeing its effects on others:

They had one here, a biopsy on their leg, I think it was. At first, [when] I thought about it, I said, “Oh, that’s easy.” But then when I saw what people are suffering, they can’t even sit down. I mean they go through that, the-the pain, and they have to be with their leg. And I saw this lady-, I said, “Oh, no, no, no, I don’t think I will go for that.”

Lena demonstrates how participants’ willingness to enrol in certain phase I trials can be changed by others’ experiences. Likewise, witnessing others’ experiences in lumbar puncture studies can confirm that these are also studies best avoided. For example, Oscar, a Hispanic man in his thirties who had participated in seven studies, had been tempted to enrol in a lumbar puncture study after his cousin had done one and used the $8,000 study compensation to buy a car. When he finally called the clinic, the study was no longer recruiting. Oscar enrolled in a different phase I trial instead, and coincidentally, he was in the clinic with a group in the lumbar puncture study. Oscar recalled,

There was actually a guy … he was doing a spinal tap. And I just happened to walk past the room and the door was open, and I saw him like laying on his side and just zoned out, and not even really reading anything or watching TV, just like staring at the wall, just like laying there on his side. And I was like, “I think that man is dead.” … So anyway … he was totally fine. But … I always tell them [the recruiters], “Oh, no, no, that’s something I would never do.”

Thus, participants’ vicarious experiences and observations profoundly shape their perceptions of these procedures’ risks, perhaps more so than the informed consent process.

### Long-Term Risks

For participants, phase I trials’ long-term risks are highly uncertain. For example, when asked about the long-term risks, Millie, a black woman in her twenties who had participated in two studies, responded,

That’s the thing, I don’t know. They say after, like, this study, the stuff should be out of your system within thirty days or something like that. I’m not sure how it actually, like if it’s attacking any of my blood cells or anything. So, it just really depends.

Information about risks that are merely speculative is generally not a required (or condoned) part of informed consent. In the absence of such information, many healthy volunteers worry about developing a health problem after a long latency period. This was true for Enrique, a Hispanic man in his forties who had participated in six studies:

And sometimes what I do think about is that maybe someday … it might cause harm in my organs, in my kidneys, or in my lungs, or in my liver or in my skin or with my eyesight or in my ears—in parts [of the body] that … are necessary for you to function properly. (*translated from Spanish*)

Concern about long-term risk was particularly acute among participants who believed a study drug could trigger the illness it was being developed to treat. Enrolled in his first study, Gavin, a white man in his thirties, articulated this worry:

I guess the only thing I’m really kind of concerned with is not short-term, it’s long-term. You know, in forty-five years, how will I feel? In forty-five years, will I have Parkinson’s because of this? So that is the only thing that has got my flag up, you know, that’s the only, really, concern I have.

Participants’ anxiety about this type of long-term risk was exacerbated when they had a family history of the disease targeted by the investigational drug. Jackie, a Hispanic multiracial woman in her forties, had mixed feelings about the diabetes drug she had taken in her first study. With several diabetic family members, she was simultaneously happy to contribute to the development of a medication that might help them and fearful that the study would negatively affect her long-term health. She confided,

Well, I just kinda think, “Okay, I’m a perfectly healthy person now, [but] … what if in like five months … I do develop diabetes?” [*laughs*] I’m like, “Oh, I was taking this drug to, you know, to help study diabetes,” and then, you know, you get it. … I kinda think about that.

In each case, the participants perceived that a single clinical trial could create health problems long after the study ended.

Participants also expressed concern about potential long-term effects from serial phase I participation. From this perspective, each clinical trial might not have long-term risks, but the accumulation of investigational drugs in multiple trials could damage one’s overall health. This is a type of risk not covered by trial consent processes, which focus on individual clinical trials, but many participants assumed that serial participation imparted long-term risks. Underscoring this point, Roscoe, a black man in his forties who had participated in five studies, observed, “I think it’d be hard on your body [to do studies long-term]. … I think the long-term effect, if you keep doing it, I think you’re taking a risk. … Eventually it can catch up with you.” Citing these potential long-term effects, Roscoe and other participants like him wanted to curb their trial participation to stave off any damage that could occur.

Creating ambiguity about long-term risks—from either a single trial or serial participation—are the bodily changes participants experience over time. Some attributed these changes to the natural process of aging, but many wondered whether study participation was the cause. Sylvester, a black man in his twenties who had participated in twenty studies, confided,

I have a concern about things. A recent concern of mine has been [that] every time I come to a study, my urination is weird. Since I’m a black male, I know prostate cancer is very, very prominent in black males, so I be having concerns about that. So, I’m planning on getting scheduled to have a prostate exam.

It is likely that none of Sylvester’s past studies had indicated that long-term prostate problems could occur; however, he personally could not dismiss outright his participation as the cause because the symptoms appeared only after he had completed several trials.

Importantly, some participants were adamant that phase I trials have no potential long-term effects. Those individuals typically justified their belief based on how briefly healthy volunteers are exposed to an investigational drug. Wanda, a black woman in her fifties who had participated in seven trials, argued,

I don’t think it’s going to affect me for the long-term … because in most cases they’re only giving you one pill, you know? … So I’m not worried about, you know, taking one pill ‘cause you got drug addicts that take drugs every day, and it doesn’t affect them for years, you know, so I don’t worry about that too much.

Interestingly, when participants denied the possibility of long-term risks, they never did so by referring to the consent process. They could, for instance, have claimed that because the official trial information they were given did not include any long-term risks, these must not be real possibilities. Instead, they defended their position by appealing to other factors about clinical trials or health risks generally, and in that way mirrored the approach of participants who *were* concerned about long-term risks.

### Therapeutic Areas

Because consent processes focus on individual clinical trials, healthy volunteers receive no information comparing the risks of different types of investigational drugs. Participants nonetheless inferred how risks might differ based on the therapeutic area under investigation, often relying on their broader impressions of the illnesses rather than experience with particular clinical trials. As we have written elsewhere (Cottingham et al. 2018), some participants perceived investigational drugs for HIV or AIDS as posing an “exceptional risk,” with deeper cultural fears about the disease inflecting their view of these phase I trials. Similar narratives about cancer studies and drugs that might affect the brain cast those therapeutic areas as requiring “strong”—and therefore riskier—medications. Focusing on cancer studies, Esteban, a Hispanic man in his thirties who had participated in ten studies, explicated, “In theory, if a medication is for cancer, it kills malignant cells. But when you kill malignant cells, you’re also going to kill good cells. That’s going to affect you, so I don’t do those types of studies” (*translated from Spanish*). Likewise, participants also identified psychotropic medications as those with profound risks to healthy volunteers. For example, Derek, a black man in his thirties who had participated in two studies, averred,

The ones I definitely won’t mess with is anything dealing with the chemicals in the brain. Anything that’s playing with your brain a lot of times is not fixable. … Like depression or anything, it does an imbalance, it’s going to mess something up, I feel. It might decrease serotonin in your brain… your brain trying to get back on track, so it’s trying to produce more [serotonin] and make it imbalanced. No.

In these instances, participants used their assumptions about an illness’ seriousness to evaluate study risks, largely to compare the various phase I trials on offer.

While certain therapeutic areas signaled to most participants the presence of a greater risk, other types of investigational drugs created more conflicted views. Pain was one such area of drug development.^1^ On one hand, participants associated pain medication with ubiquitous over-the-counter (OTC) drugs that are popularly viewed as very safe. In this vein, Luke, a black man in his thirties who had participated in six studies, described the migraine trial in which he was enrolled: “It’s no risk. … I don’t think it’s risky ‘cause it’s like taking a Tylenol [acetaminophen], so a Tylenol ain’t risky, you know?” Participants often presumed similarities between the investigational drug and OTCs, even when molecularly the drugs had nothing in common. This manifested for Becca, a white woman in her thirties who had participated in nine studies. Believing an investigational drug for rheumatoid arthritis—a systemic autoimmune disease—was similar to OTCs used for osteoarthritis—a chronic joint disorder, Becca defended her decision to enrol in the trial: “I can justify it as it’s all kind of, you know, like Advil [ibuprofen] or Tylenol [acetaminophen]. It’s just a higher grade and a higher dosage kind of an idea. A similar, similar product.” In other words, participants like Luke and Becca concluded these study drugs were low risk by comparing them to OTC products commonly used to treat pain. On the other hand, some participants associated pain medication with addiction, which heightened their risk perception. Voicing this view directly, Carly, a Hispanic American Indian woman in her forties who was enrolled in her first study, stated,

I’d think I would be afraid to take, like, anything having to do with pain—pain pills or anything to that nature—because I know you can become dependent on it and there can be an addiction involved, so I would be afraid to do that.

These divergent views about pain medications underscore how participants evaluate phase I trial risks through the lens of their own background assumptions. Becca’s view specifically illustrates the problematic effect of misperception when participants minimalize trial risks by conflating radically different illnesses. This misunderstanding occurred despite an informed consent process that provided her with detailed information about the study. Most salient to her, and likely others in the same trial, was the notion that “arthritis” is a common and unalarming illness, unlike perhaps cancer or HIV.

### Dose Level and Frequency

As they considered phase I risks, participants also focused on the dose of investigational drugs. Rather than distinguishing among drug types, many participants believed that milligrams provide a meaningful and universal metric for risk information, with low doses as safe and higher doses as always riskier. Sylvester, the participant with prostate concerns, privileged dose in his decision-making about study enrolment:

I turned down studies before because of the milligrams … If it’s past 500 [milligrams], I don’t really do it. … I think my first study ever … was 1,000 milligrams, and I felt headaches and stuff, and I was like, it’s not worth it.

Assessing risk by dose alone appears reasonable, especially as a means of comparing phase I trials based on seemingly standardized information across consent processes. Yet, notwithstanding Sylvester’s personal experience, the relationship between dose and risk for different investigational drugs is complicated by the fact that some drugs are dangerous at very low doses whereas other drugs are safe at very high doses.

Participants’ risk perceptions were shaped not only by the amount of drug given in each dose but also by the total number of doses of an investigational drug a phase I protocol required. Many participants assumed that a single dose presented less risk than repeated exposure. Indeed, Jean, a white woman in her fifties who had participated in three studies, declared, “The first study [I did], we had one dose, so I didn’t think, really think anything would come of that.” With larger numbers of pills consumed, participants worried more about the risk. For instance, Harry, a multiracial man in his twenties who had participated in seven studies, ruminated,

This one [clinical trial] I’m doing now [with daily dosing] seems to be the most riskiest study I’ve ever done. So, if you have to take lots of drugs, take them frequently and over a long period of time, that’s more risky.

When comparing phase I trials, exposure to the drug over time might be more important risk information than the amount of the drug. Notably, the death and serious injury that occurred to healthy volunteers in a 2016 pain study in France was later attributed to repeated dosing of the investigational drug (Enserink 2016). At the same time, this perspective generally places disproportionate weight on dosing relative to other important indicators of trial risks.

### Compensation Level as Proxy for Risk

Healthy volunteers also commonly use study compensation as a comparative gauge of trial risk (Cryder et al. 2010). Among our participants, this was, for example, Harry’s primary metric for determining how risky a study might be:

The more they pay you, presumably the riskier it is. That’s kind of a rule of thumb, I would say. If they’re paying me $10,000 for a study, why are they paying so much, you know? That’s the first thing that would come to mind.

Sherrie, a white woman in her thirties who had participated in two studies, echoed Harry’s point but applied it to a study that paid $4,000 in which she had actually enrolled:

There were some serious side effects with this one. It can be a carcinogenic, so it can cause cancer. It can … cause strokes, heart attacks. … Although the bigger side effects weren’t as, the percentage wasn’t as high. But for the smaller side effects—like loss of appetite, headaches, stomachache, things like that, and redness at the injection site—probabilities were pretty high for that. … I mean, … they’re paying you this much money [i.e., $4,000] for a reason. You know what I mean? … So, you should think about that.

Clinical trials are not supposed to compensate for risk. Instead, payment should be based on time and inconvenience (Gelinas et al. 2018). This means that the longer a study is, the more it will pay participants. Longer studies also typically require more drug doses, so even when participants understand that compensation is tied to study length, this cannot be separated from their greater exposure to an investigational drug and therefore—directly or indirectly—risk. Moreover, there is no industry standard for payments allowable to healthy volunteers (Dickert, Emanuel, and Grady 2002), and there is significant variation in clinical trial payments. That study participants, rightly or wrongly, view studies that offer greater compensation as higher risk signals ongoing problems both in the ethical oversight of payment and how compensation is explained in the informed consent process.

### Risk Mitigation by Clinic

Many healthy volunteers argued that despite the numerous features that determine a phase I trial’s risks, the system of study oversight ultimately keeps them safe. Participants cited how being confined to a research clinic so they can be monitored protects them from any serious harm. Representing this view was Lena, the participant who would refuse to participate in a biopsy study:

That’s why they keep us in-house, so they can monitor on a daily base [*sic*], on a hourly base [*sic*] when we have the medicine. It’s like every half hour they draw blood, they do EKGs and [blood] pressure. So, they’re monitoring us really good in the first 24 hours once we dose. So I feel—myself—I feel that it’s pretty safe.

Participants believe that monitoring keeps them safe because they assume that researchers can handle any urgent medical problems that occur. Some participants, like Garth, a white man in his thirties who was enrolled in his second study, undoubtedly had too much faith in the monitoring process: “I’m sure … they’re not going to put people in major jeopardy. I mean if I’m allergic to something, they have the, you know, the drug to counteract that, which is, thank God, a good thing, you know?” Many also emphasized that it is not in researchers’ best interest to have healthy volunteers die in a clinical trial. For example, Jervis, a black man in his thirties who had participated in four trials, remarked,

[Phase I trials] have some risks, but they are very minimum because they [the researchers] themself don’t want to, you know, kill you in any way. If they do, that’ll be, you know, bad news. But, they are all precautions taken. … You find they are professionals.

By adopting this view of phase I clinics, some healthy volunteers might be more comfortable in enrolling in higher risk studies because they trust that researchers will protect them from serious harm.

## Discussion

Previous research has documented that healthy volunteers perceive clinical trial risk on a spectrum between very risky to quite low risk (Chen et al. 2017; Fisher et al. 2018). Focusing on participants’ measure of trial risks, however, has offered limited insights into how they actually make those assessments. Our study illustrates the diversity of factors that contribute to healthy volunteers’ understandings of trial risks, such as formal trial information, participants’ own and others’ experiences in clinical trials, comparisons among studies, and levels of trust in the research enterprise. It also shows how the informed consent process both provides concrete information and creates lacunae about the risks of particular trials.

Our findings support previous studies that highlight how consent forms and trial information sheets are best suited to communicating particular types of information. Specifically, consent documents lend themselves well to describing the type of trial and the procedures involved (Berg et al. 2001), such as the volume of blood collected and on what schedule. They also appear to be effective at describing short-term risks, such as the likely side effects that might result from consuming the investigational drug (Corrigan 2003). In our study, participants seemed to understand well and make enrolment decisions based on the temporary side effects or medical procedures associated with a study.

Despite its robustness, the consent process also allows certain misunderstandings about phase I trial risks to foment (see also Sankar 2004). Importantly, these misunderstandings serve in some cases to diminish participants’ perceptions of risk and in others to amplify them. We found evidence of the former in participants’ view that low doses of investigational drugs were determinative of a safe study regardless of the chemical makeup of each drug. Additionally, participants might not fully appreciate that some trial harms cannot be managed or reversed by the research team, meaning that the risks are greater than these individuals believe. Exacerbating risk misperceptions was the tendency to view greater study payments as compensation for exposure to higher risk, even though many such studies merely took longer to complete. Risk perceptions were affected in both directions when participants judged study risks based on preconceived notions about the seriousness of the illness for which the investigational drug was being developed. This led in some cases to participants exaggerating the risks of a drug associated with cancer or HIV and in other cases to participants underrating the risks of pain medication. Likewise, unusual procedures, such as biopsies and lumbar punctures, often directed participants’ attention away from the investigational drug so that the study’s risks were decided by their view of those procedures alone.

Finally, healthy volunteers felt tremendous uncertainty about the long-term risks of phase I trial participation. This manifested in participants’ anxiety that consuming an investigational drug could trigger the disease it was targeting as well as in their unanswered questions about the cumulative effects of serial participation. Whether or not their anxiety was created by the consent process, that process clearly did not dispel it. Phase I trial consent forms and trial information sheets often provide scant information about long-term risks. Participants might find the absence of formal warnings about long-term risks as indicative that the information is missing rather than researchers’ expectation that long-term problems are unlikely or unknown.

In general, the ethical requirements for consent centre on the duties involved in informing individuals about discrete study participation choices (Sreenivasan 2003). In phase I healthy volunteer trials, however, participants compare the risks of diverse trials in deciding when to enrol. Because such information is not part of the consent process, healthy volunteers must adjudicate for themselves with partial and inaccurate information how the risk of one available phase I trial might compare to another. That healthy volunteers do so is evident not only from our study but also prior research on this population that shows how individuals make different types of decisions about their study participation (Grady et al. 2017; Roberts and Kim 2017; Rabin and Tabak 2006).

Our study adds to this literature by revealing that some risk misperceptions are encouraged by the structure of phase I trials. Because healthy volunteers are generally not seeking particular studies, the varied offerings can encourage them to compare studies in order to maximize how much they earn, on one hand, and minimize the risk to which they are exposed, on the other. However, the heterogeneous nature of phase I trial risks complicates this process. For example, despite efforts to ensure that phase I trials are generally low risk, some drugs are in fact dangerous at low doses and others are safe at very high doses. Additionally, the confinement structure of phase I trials encourages the sharing of risk information among participants. This means that participants assess risks based in part on what adverse effects they witness during studies or what other participants tell them about different studies’ risks. These informal information sources might even compete with the formal consent process, leaving participants to speculate, in particular, about risks and safeguards not explicitly addressed by the consent process.

Because the informed consent process largely ignores many of the background assumptions that healthy volunteers use to make sense of formal trial information, we propose additional consideration of the following ethical factors regarding the consent process: financial inducements, bidirectional communication about study risk, and addressing unknown risks.

### Financial inducement

The U.S. system of clinical trial oversight allows payment for research participation provided it does not unduly influence decision-making. Nonetheless, monetary incentives are not considered benefits that may offset risk in the ethical evaluation of clinical trials (U.S. National Institutes of Health 2005; U.S. Food and Drug Administration 1998). Doing otherwise would be ethically problematic, especially for trials that carry significant medical risks. However, as we have illustrated, healthy volunteers do balance risk against financial benefit in their decision-making about phase I trials. Therefore, ethics review boards and phase I investigators must be realistic about the extent to which participants’ risk perceptions might be transformed by financial benefits. Furthermore, it is important to recognize that even though study compensation is largely set to incentivize consent to clinic confinement, individuals considering enrolling are likely to tie higher amounts directly to greater risks. These perceptions ought to be addressed explicitly in the consent process.

### Bidirectional communication about study risks

Healthy volunteers hold complex and varying views about how risks of phase I trials are constituted. These views may be more or less scientifically plausible, and as we demonstrated, they may also be shared or witnessed within the community of participants and/or idiosyncratic to individual participants. In describing trial risks during the informed consent process, researchers must be aware of the risk perceptions, including study procedure risks, that each participant brings to the conversation so that researchers can better engage with these perspectives. Data from our study offer specific content areas that researchers and healthy volunteers could more fully discuss. For example, researchers could clarify for participants that a lesser dose of a drug should not necessarily be conflated with a lower risk. In addition, researchers could ensure that participants understand how certain potential harms cannot be ameliorated, even by attentive clinic staff.

### Unknown or unknowable risks

Although the risk of any phase I trial will be somewhat uncertain, many healthy volunteers express concerns about the longer-term impact of an investigational drug as well as of serial trial participation. As we have already noted, these risks are plagued with even more ambiguity as little information or evidence exists to anticipate long-term harms. However, investigators and ethics review boards could structure the informed consent process to address concerns that emerge from healthy volunteers’ serial trial enrolment. This would mean contending with participants’ diverse experiences with and conjectures about more speculative forms of risk. More broadly, our study also suggests that the informed consent process for all clinical trials should include more explicit discussions with prospective participants about the likelihood and nature of long-term or cumulative risks.

## Conclusion

Our intention here is not to suggest that phase I clinics have been remiss in their duty to secure informed consent from healthy volunteers, but our findings do underscore the importance of empirical research on trial participants’ perceptions of risk. Participants are neither passive recipients of risk information nor fully knowledgeable actors when it comes to making risk-related decisions; rather, their risk perceptions are constantly constructed, maintained, and negotiated within a larger community of serial participants and a culture of clinical research. Personal experiences and observations can significantly influence risk perceptions and decision-making, but these are integral parts of the context for phase I research, not separate corrupting factors.

The particular types of misunderstandings that healthy volunteers have about trial risks are not obvious, and some of these can be addressed in the broader informed consent process if not through the documents that prospective participants receive for particular trials. Some scholars have argued for shorter, more streamlined consent forms and trial information sheets in phase I trials (Stunkel et al. 2010), but the problem appears to stem not from providing too much information but instead fine-tuning what details might be particularly important. Given that healthy volunteers express ambivalence about the long-term participation risks, more can be done during the consent process to explain how and why long-term effects might occur or, when relevant, are impossible or unlikely. Likewise, knowing that participants are apt to make risk and financial benefit comparisons among different phase I trials, the consent process should debunk false assumptions about drug dose and therapeutic class as well as address participant perspectives about the tie between financial compensation and trial risk. Additionally, when invasive procedures are required in trials, the risks of the drugs related to those procedures (e.g., blood clotting times for biopsies or permeability of the blood–brain barrier for lumbar punctures) must be emphasized more. Finally, in order to correct unrealistic views of the safety provided by clinics, staff should be forthright about the purposes for and limitations of monitoring participants’ reactions to investigational drugs. The informed consent process can be improved, and part of the ethical duty to meet participants where they are requires that we understand the basis for their beliefs about risks and help them make decisions that are grounded in the most accurate information possible.

## Footnotes

1 These interviews were conducted in 2013 prior to the extensive media coverage of the U.S. opioid epidemic. It is possible that participants today would have different views of pain medication as a result of more information about the use of addictive narcotics to treat pain.
