# Correction to: Picking and Choosing Among Phase I Trials

**DOI:** 10.1007/s11673-020-10031-w

**Published:** 2020-08-28

**Authors:** Jill A. Fisher, Torin Monahan, Rebecca L. Walker

**Affiliations:** 1 Department of Social Medicine and Center for Bioethics, University of North Carolina at Chapel Hill, CB 7240, Chapel Hill, NC 27599-7240 USA; 2 Department of Communication, University of North Carolina at Chapel Hill, CB 3285, Chapel Hill, NC 27599-3285 USA

**Correction to: Bioethical Inquiry (2019) 16:535–549**

10.1007/s11673-019-09946-w

The article "Picking and Choosing Among Phase I Trials", written by Jill A. Fisher, Torin Monahan and Rebecca L. Walker, was originally published Online First without Open Access. After publication in volume 16, issue 4, page 535-549 the author decided to opt for Open Choice and to make the article an Open Access publication. Therefore, the copyright of the article has been changed to © The Authors 2019 and the article is forthwith distributed under the terms of the Creative Commons Attribution 4.0 International License, which permits use, sharing, adaptation, distribution and reproduction in any medium or format, as long as you give appropriate credit to the original author(s) and the source, provide a link to the Creative Commons licence, and indicate if changes were made. The images or other third party material in this article are included in the article’s Creative Commons licence, unless indicated otherwise in a credit line to the material. If material is not included in the article’s Creative Commons licence and your intended use is not permitted by statutory regulation or exceeds the permitted use, you will need to obtain permission directly from the copyright holder. To view a copy of this licence, visit http://creativecommons.org/licenses/by/4.0/

